# Identification of Safety and Quality Parameters for Preparation of Jellyfish Based Novel Food Products

**DOI:** 10.3390/foods8070263

**Published:** 2019-07-17

**Authors:** Gianluca Bleve, Francesca Anna Ramires, Antonia Gallo, Antonella Leone

**Affiliations:** National Research Council, Institute of Sciences of Food Production, Lecce 73100, Italy

**Keywords:** edible jellyfish, novel food, safety, quality, bacteria, yeasts and molds

## Abstract

Edible jellyfish are mainly consumed and marketed in Southeastern Countries, generally produced by a multi-phase drying process, using mixtures of salt and alum. Recently, jellyfish have become very attractive also for Western food markets. They are novel food in Europe and no recognized handling/processing steps have been set up yet. Moreover, no specific food safety and quality parameters are available. In this study, we identified a set of safety and quality parameters for jellyfish, based on standards and process hygiene criteria used in Europe for other products. These assays were tested on three different jellyfish preparations that can be used as raw materials for subsequent food processing. All jellyfish samples revealed the absence of pathogens (*Salmonella* spp. and *Listeria monocytogenes*), *Enterobacteriaceae* and *Pseudomonas* spp., even if a limited presence of *Staphylococci* was observed. No biogenic amine histamine was detected and negligible levels of total volatile basic nitrogen (TVB-N) were revealed. Total bacterium, yeast and mold counts were negligible or undetectable by conventional accredited methods, and conversely the results were higher when optimized saline conditions were used. This study, for the first time, established a set of quality and safety parameters necessary for first-operations and subsequent processing of jellyfish as novel food. Highlights: Jellyfish can represent a novel food in Europe. Identification of safety and quality parameters for jellyfish food products. Saline conditions are essential for improving safety and quality assessment of jellyfish as food.

## 1. Introduction

In Southeast Asia, the fishery and market of edible jellyfish (JF) is around 1 million tons of JF landings and the corresponding estimated business exceeds 100 million US $ [1]. The main producers of edible JF are China, Indonesia and Malaysia, even though new species from the USA, Mexico, Australia and India have been exploited and used for food preparations and exported to Asia [2,3,4]. Currently, active JF fishing is carried out in at least 19 countries [1] with many companies involved in the production and import-export of edible JF [5].

Very recently, the Food and Agriculture Organization of the United Nations (FAO) has considered that the introduction of JF as new seafood products for human consumption, and as feed for aquaculture, can represent a possible strategy to improve the overall sustainability of the global fishery sector [6]. Indeed, an increased frequency and abundance of JF outbreaks has been reported in the Mediterranean and North Sea [6], affecting fishing and sea wildlife with evident economic impacts [7,8,9]. Meanwhile, recent research studies have reported that some JF species with outbreak-forming populations in the Mediterranean Sea have biochemical and textural features similar to edible Asiatic species [10,11,12]. These native JF species in the Mediterranean Sea seem to be good candidates as a new “local seafood product” characterized by nutraceutical features [10,13].

In Europe, the lack of food tradition makes edible JF market still virtually absent or possibly restricted to the Asiatic immigrant community. Currently, JF and JF-derived food products are not explicitly mentioned in the European regulation on microbiological criteria for foodstuffs [14,15], even though JF are included in the Codex Alimentarius [16] among aquatic invertebrates in the Food Category descriptor 09.0 “Fish and fish products, including molluscs, crustaceans, and echinoderms”. In the absence of significant consumption, JF are labelled as “novel food” under the current European Regulation [17], which contains specific guidelines for traditional foods in third Countries. According to this regulation, the marketing of JF can be approved following a request for a notification or authorization. The recent upgrade of the EU rule on novel foods, the globalization of food markets and the increased availability of JF local biomass open up new possibilities for the introduction of edible JF in the diet of European citizens.

Commercially exploited JF belong to the phylum Cnidaria, class Scyphozoa and particularly to Rhizostomeae species of the genera *Rhopilema*, *Nemopilema*, and *Stomolopus* [2,11,18]. Asiatic edible JF are produced by the Asiatic traditional method, which consists of dehydrating JF using a mixture of salts and alum to stabilize the product and to obtain the expected taste and texture traits [2,19,20]. However, they are poorly characterized for their quality and safety aspects and nutritional traits by standard methodologies. The possible introduction of JF consumption as food in European Countries requires a report based on the history of safe food use in a third country or the development of new JF processing methods for safe food use of JF. This last option could be mandatory for some Mediterranean JF species, which are still not considered edible such as some Mediterranean species. A safety issue in the traditional Asiatic JF processing is related to the use of alum as a firming agent due to its negative effects on human health [21]. Then, an alternative process has to be designed to obtain final products observing safety and quality standards, maintaining nutritional traits, having sensory properties suitable for Western consumers. This is particularly true for raw material that has no history as food in western Countries and specifically in the European area [22].

At the present time, a production chain both for edible JF and JF-derived foods does not exist in Europe and handling/processing steps together with associated critical control points have to be determined.

In this study, the main safety and quality parameters suitable for the future risk assessment of JF, as a novel food in Europe, were identified. These parameters were tested on three different JF preparations that can be used as raw materials for subsequent food processing. The barrel JF, *Rhizostoma pulmo*, from the Ionian Sea Italian coasts has been assessed as model for human food preparations alternative to traditional ones. Moreover, the necessity to develop new specific and dedicated assays for JF and JF-derived products, mainly due to the possible presence of pathogens and spoilage microbiota and/or possible toxic metabolites, has been here demonstrated.

## 2. Materials and Methods

### 2.1. Sample Collection and Preparation

**Figure 1:**
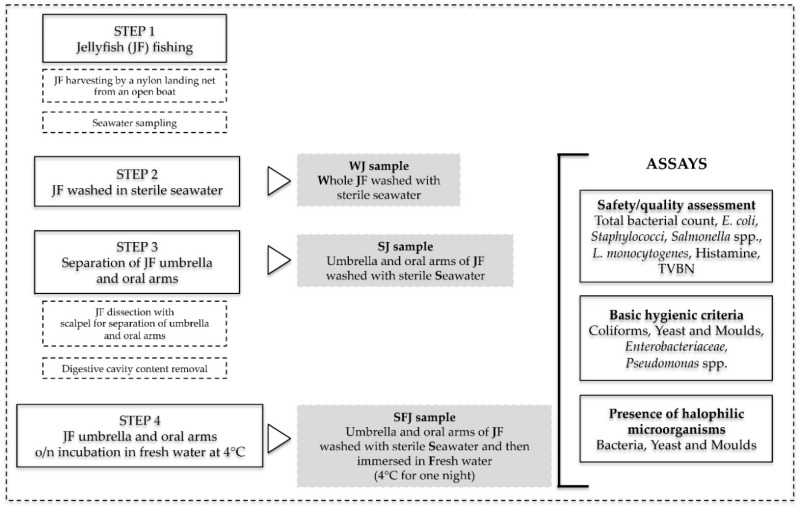
Diagram illustrating samples collection, preparation and analyses. TVBN: total volatile base nitrogen. WJ: whole jellyfish washed with sterile seawater. SJ: umbrella and oral arms of jellyfish washed with sterile seawater; SFJ: umbrella and oral arms of jellyfish washed in sterile seawater and then immersed in fresh water at 4 °C for one night.

Specimens of jellyfish (JF) species *Rhizostoma pulmo* were collected offshore in Ionian sea (Ginosa Marina, Italy 40°41′2117″ N; 16°88′0284″ E) in four sampling periods (June, July, August and October 2018), with a nylon landing net with 3.5 cm mesh size, from an open boat. Three different types of samples were produced (Figure 1): 1) whole JF washed with sterile seawater (WJ), umbrella and oral arms of JF washed with sterile seawater (SJ), umbrella and oral arms of JF washed in sterile seawater and then immersed in fresh water at 4 °C for one night (SFJ). WJ samples were deposited in sterile plastic bags in a refrigerated container on ice and stored in a shaded place during boat operations. For SJ preparation, immediately after fishing, JF were immersed in sterile seawater and dissected with a sterile scalpel for separation of the umbrella and legs. The content of digestive cavity (gonads and fluid gastric content) was removed by scraping the umbrella under the inner surface using a sterile scalpel and collected with a sterile syringe in 50 mL sterile Falcon tubes and kept on ice for further analyses. The umbrella and oral arms were washed three times with sterile seawater to remove transient and/or loosely associated microorganisms from the surfaces. Samples were stored in sterile plastic bags on ice. The organisms were transported to the laboratory within two hours after collection and/or treatment. For SFJ preparation, samples treated as SJ were then soaked in sterile freshwater and incubated at 4 °C for one night. Samples of seawater (C) were collected at each sampling, as control.

### 2.2. Microbiological Analyses

All the three types of JF samples were delivered to the external accredited laboratory (Laboratori Artas Società cooperativa, Poggiardo, Lecce, Italy) stored in sterile plastic bags on ice in a box. The accredited laboratory performed the analyses, with the exception of assays for enumeration of *Enterobacteriaceae* and *Pseudomonas* spp.

Ten grams of each sample were added to 90 mL of Buffered Peptone Water (Biolife Italiana, Milano, Italy) as diluent (1:10) and homogenized for 2 min in a Stomacher, in accordance with specific standard methods for total bacterial count [23], coliforms [24], β-glucuronidase positive *Escherichia coli* [25], coagulase positive *Staphylococci* [26], yeast and molds [27,28]. For the detection of the pathogenic bacteria *Salmonella* spp. [29] and *Listeria monocytogenes* [30], 25 g of jellyfish samples were suspended in 225 mL of Buffered Peptone Water (Biolife Italiana, Milano, Italy) and Fraser Broth Half concentration (FBH, Biomerieux, Marcy l’Etoile, France), respectively, as diluent.

For the total bacterial count (TBC), samples were diluted and plated by pour plate technique on Plate Count Agar (PCA) (Biolife Italiana, Milano, Italy) pH 7.0 and incubated at 30 °C for 72 h; enumeration of yeast and molds were performed by incubation at 25 °C for 5 days on Dichloran Rose–Bengal Chloramphenicol Agar (DRBC, Thermo Fisher Scientific, Monza, Italy). The presence of coagulase-positive *Staphylococci* was checked on Baird Parker Agar with Rabbit Plasma Fibrinogen (RPF, Thermo Fisher Scientific, Monza, Italy) supplement at 37 °C for 24 h, whereas diluted samples were plated by pour plating method on ColiID Agar (Biomerieux, Marcy l’Etoile, France), and incubated at 37 °C for 24 h for the enumeration of coliform bacteria (blue colonies) and *E. coli* (red colonies).

Determination of *Salmonella* spp. was performed according to UNI EN ISO 6579:2017 methods [29]. The starting material was firstly pre-enriched on diluent at 37 °C for 18 h, then it was subjected to a combined process of selective enrichment in Rappaport Vassiliadis Salmonella (RVS) broth (Thermo Fisher Scientific, Monza, Italy) at 41.5 °C for 24 h, as well as Mueller Kauffman Tetrathionate (MKTT) broth (Thermo Fisher Scientific, Monza, Italy) at 37 °C for 24 h. Aliquots of samples deriving from RSV and MKTT broth were streaking plated on *Salmonella Shigella* (SS) agar (Thermo Fisher Scientific, Monza, Italy) and Xylose-Lysine-Desoxycholate (XLD) Agar (Thermo Fisher Scientific, Monza, Italy) at 37 °C for 24 h. Colonies grown on these media were streaked on nutritive agar at 37 °C for 24 h and finally identified by API (Biomerieux, Marcy l’Etoile, France) and specific antiserum test.

*Listeria monocytogenes* was identified by ISO 11290-1:2017 method [30]. According to this protocol, samples suspended in diluent, were incubated at 30 °C for 24 h, and then widespread on ALOA Gelose Agar (Biomerieux, Marcy l’Etoile, France) and subsequently incubated at 37 °C for 48 h.

Further microbiological analyses were applied by testing diluted samples in peptone water on agar slants containing the following media: Violet Red Bile Glucose Agar (VRBGA, LABM, Heywood, UK) was used for *Enterobacteriaceae* enumeration by incubation at 37 °C for 18–24 h; *Pseudomonas* Agar Base (LABM, Heywood, Lancashire, UK) supplemented with Cetrimide, Fucidin, Cephaloridine (CFC) selective agar supplement (LABM, Heywood, Lancashire, UK) was used for *Pseudomonas* spp. identification by incubation at 30 °C for 18–24 h.

### 2.3. Chemical Analyses

The total volatile base nitrogen (TVBN) was determined by treating each jellyfish sample (100 g) with 0.6 M perchloric acid (Merck KGaA, Darmstadt, Germany). After alkalization, the extract was exposed to steam distillation and an acid receiver absorbed the volatile base components. The TVBN concentration was determined by titration of the absorbed bases following the method EC Regulation N. 2005/2074 [31].

Histamine concentration was determined according to the method AOAC N° 021402 (2014) (HistaSure ELISA, LDN, Nordhorn, Germany) [32].

### 2.4. Determination of Halophilic Microorganisms

JF samples were homogenized with a sterilized blender and 25 g of each sample were added to peptone seawater (0.1% w/v peptone, artificial seawater (3% NaCl, 0.07% KCl, 1.08% MgCl_2_, 0.54% MgSO_4_, 0.1% CaCl_2_, w/v [33]). All samples and their respective serial dilutions were plated in different media dissolved in artificial seawater: saline PCA (sPCA, LABM, Heywood, Lancashire, UK), R2A (Sigma-Aldrich, Darmstadt, Germany), Marine Agar (peptone 5 g/L, yeast extract 1 g/L, agar 16 g/L) added with 0.05 g/L of nystatin and incubation at 30 °C for 48–72 h for bacteria or added with 0.1 g/of ampicillin and 0.05 g/L of kanamycin and incubated at 25 °C for 2–7 days for fungi; saline Sabouraud Dextrose Agar (sSDA, LABM, Heywood, Lancashire, UK) and saline Corn Meal Agar (sCMA, Sigma-Aldrich, Darmstadt, Germany) added with 0.1 g/of ampicillin (Merck KGaA, Darmstadt, Germany) and 0.05 g/L of kanamycin (Merck KGaA, Darmstadt, Germany). For each plate, the number of colony forming units (CFU) per gram of JF was determined.

### 2.5. Statistical Analysis

All data represent the mean of three independent replicates (*n* = 3).

Statistical analysis was based on one-way analysis of variance. Tukey’s post-hoc method was applied to establish significant differences among means (*p* < 0.05). All statistical comparisons were performed using Sigma-Stat, version 3.11 (Systat Software Inc., Chicago, IL, USA).

## 3. Results

The first step of this study was to individuate the most important quality and safety parameters, in compliance with European Union laws, to be applied to JF-based foods. To do this, the law in force in terms of food safety and process hygiene criteria were analysed. Selected standards establish limits for microbial pathogens such as *Salmonella* spp., *E. coli*, coagulase-positive *Staphylococci* count, the biogenic amine histamine and the content of TVBN (Table 1). In this study, other supplementary safety and hygiene process criteria were selected from those already established by the law in force in Italy [34] and in France [35] for fish and fish-derived foods, molluscs and crustaceans. All the parameters considered in the current study are listed in Table 1. They were applied to analyse different types of *R. pulmo* samples collected on four different occasions off the Ionian coast during June-October 2018.

Three different preparations of JF, chosen as possible starting material for setting up subsequent food processing methods, were considered (Figure 1): (1) whole JF (WJ); (2) JF umbrella and oral arms washed with sterile seawater (SF); (3) JF umbrella and oral arms washed with sterile seawater and then immersed overnight in fresh water at 4 °C (SFJ). These samples were tested for the presence of possible pathogenic and spoilage microorganisms. Samples of seawater (C) collected during JF sampling were also assayed as control in order to evaluate the environmental microbial count.

As shown in Table 2, in all JF samples, pathogens *Salmonella* spp. and *L. monocytogenes* were not detected in all the four sampling times (June-July-August-October 2018). Total counts of *Staphylococci* showed a similar trend in all water and JF samples. Indeed, they ranged from 0 to 1.4 × 10^2^ CFU/g in the untreated samples (WJ), from 0 to 3.7 × 10^2^ CFU/g and from 0 to 2.6 × 10^2^ CFU/g in SJ and SFJ samples, respectively. These data can be correlated to the *Staphylococci* presence in seawater samples (C), where total counts ranged from 0 to 1.5 × 10^2^ CFU/g. *Escherichia coli* and coliforms were undetectable in WJ, SJ and C samples during the entire period of sampling. Relatively low level of *E. coli* and coliforms contamination were detected in SFJ. It was up to 5.5 × 10^1^ CFU/g and up to 7.7 × 10^2^ CFU/g, respectively. However, TBC in all JF samples and in all sampling times ranged from 2.7 × 10^1^ CFU/g to 8.5 × 10^2^ CFU/g. Yeast and moulds were undetectable in all JF and seawater samples. In addition, no histamine was revealed in any tested samples during the samplings, revealing that microorganisms detected in some samples were not able to produce this biogenic amine in these conditions. In our study, TVBN ranged between 0.22 and 1.39 mg/100 g in WJ samples, between 0 and 1.39 mg/100 g in SJ samples and between 3.51 and 5.56 mg/100 g in SFJ samples. Among all microbiological and chemical parameters applied to the different JF treatments and to seawater samples along the four sampling times, statistically significant differences were observed only for TVBN values (Table 2). However, all TVBN values observed in JF samples were very below the limits established for products suitable for human consumption, ranging from 25–35 mg/100 g [31].

These analyses revealed that JF (data refer to *R. pulmo* species and to the period of time considered in this study) did not contain common pathogens, except for *Staphylococci* in few cases, which can probably derive from the specific marine environment. Moreover, no quality loss was detected since TVBN was very low in all samples. In addition, the absence of *Enterobacteriaceae* and *Pseudomonas* spp. was verified in all JF and in seawater samples.

As a further step, the possible presence of halophilic/halotolerant microorganisms associated to JF samples were tested on saline media (added with artificial seawater). Total counts of bacteria, yeasts and molds on saline media are reported in Table 3.

In saline PCA medium, total bacteria counts were almost similar to the values observed using the conventional accredited method. Instead, the counts in R2A and Marine Agar media resulted at least one order of magnitude higher than the corresponding values for total bacteria in non-saline medium (Table 2). However, in saline media, a statistically significant reduction of the bacteria levels was revealed, from 2.5 × 10^2^–4.5 × 10^3^ CFU/g to 2 × 10^1^–6.2 × 10^2^ CFU/g moving from WJ to SFJ samples, respectively.

The most interesting results regarded the presence in saline media of higher counts of yeast and molds. This evidence was in contrast with data obtained by conventional accredited method applied to the same samples. Also in this case, the treatments on JF biomass (SJ and SFJ) were useful to produce a statistically significant reduction of the presence of yeast and molds from 9.8 × 10^1^–1.5 × 10^3^ CFU/g in WJ samples to 2.8 × 10^1^–5.5 × 10^1^ and 0–2.5 × 10^1^ CFU/g in SJ and SFJ samples, respectively (Table 3).

## 4. Discussion

The first goal of this study was the identification of a set of main relevant quality and safety parameters to be applied for the possible consumption of JF-based foods in Europe. In the absence of specific indications, we considered market safety standards in force in Europe for food products comparable to JF [14,15,31], together with diverse basic hygienic criteria valid for fish and fish-derived foods in some European Member States [34,35].

These parameters worked efficiently when applied to different steps of JF preliminary treatment, traditionally used in Asiatic processing methods [20]. Indeed, JF are extremely perishable raw materials, and are generally treated within a few hours of harvesting in order to avoid spoilage. Moreover, the efficacy of this approach was demonstrated by the absence or very limited presence of already known possible microbial contaminants in all JF tested samples. The low level of *Staphylococci* detected in all tested JF samples and in seawater may be attributable to hygienic conditions and pollution of marine environment, since these microorganisms are generally from both human and animal sources.

In this study, the direct application of accredited available microbiological and chemical assays seems to reveal JF cannot represent a source of severe microbiological hazards for humans. Recently, Raposo et al. have reported similar evidence by applying some safety important parameters, such as *Salmonella* spp., *Listeria monocytogenes*, *Vibrio* spp. and *Aeromonas hydrophila* tests, together with heavy metal contaminants and allergenic assays on raw material and umbrella product snack derived by edible JF *Catostylus tagi* [39].

The need to increase knowledge on JF applicability as novel food for humans can be demonstrated by the presence of other potential microbiological safety hazards associated with them, such as discrete counts of microorganisms with halophilic/halotolerant characteristics not revealed by the regulated criteria and counting methods for foods in force in Europe. Although preliminary in their application, the saline media can be useful in the detection of both bacteria and yeast and molds during treatment steps of JF derived products. Even though traditional culture-dependent methods, generally applied in accredited methods, provide information regarding only the cultivable part (1–5%) of the total microbial community [40], this last one can be considered representative of that obtained from a culture-independent approach [41].

Fungi belonging to different genera *Aspergillus*, *Cladosporium*, *Purpureocillium*, and *Tilletiopsis* were isolated as JF-associated microorganisms in *Nemopilema nomurai* [42], whereas the possible pathogenic activity, in terms of toxins produced by endobiotic bacteria in JF species *Cyanea capillata* and *C. lamarckii*, has been suggested by Schuett and Doepke [43]. The presence of possible endosymbiontic bacteria has also been suggested in tissue of *Aurelia aurita* [44]. However, at the present time, the actual relationship between JF and bacteria (*Stenotrophomonas* spp., *Alteromonas* spp., *Pseudoalteromonas* spp. and *Vibrio* spp.) in terms of tissue residence has not been determined [41,45]. At the moment, studies on the interaction, persistence and metabolic activities of these microorganisms in JF and JF-derived novel foods are not available. Moreover, only few examples characterizing secondary metabolites produced by JF-associated actinomycetes and fungi have been produced [46,47,48,49]. This study demonstrates that new tools for safety and quality assessment of this novel food are necessary in the next future.

Experiments are now in progress for the identification and characterization of bacteria and fungi isolated from JF together with their possible toxic or beneficial metabolites, in order to discover their possible negative (i.e., new pathogens) or positive (i.e., probiotics) role for humans. Future studies should consider metagenomics and metabolomics approaches for the analysis of raw and processed JF for obtaining information on total microbiota associated to JF and qualitative and quantitative data on microbial metabolites.

*Rhizostoma pulmo* (known as sea lung or barrel JF) was used here as model organism, since it is considered edible in some Asian countries [20] and it has also been recently considered one of JF species in setting up a possible new method to obtain a potential human food or food ingredients for European Countries [11,12,13]. The set of parameters proposed in this study should be applied and validated also for other abundant and putatively edible JF species in Mediterranean Basin (i.e., *Aurelia aurita*, *Cotylorhiza tuberculata, Rhopilema nomadica*, etc) suitable to be used as novel foods in Western countries.

## 5. Conclusions

In this study, a collection of quality and safety parameters together with “process hygiene criteria” were identified and applied for assessment of JF as novel food in Europe. The results here reported indicate the importance of basic JF treatments by separating the umbrella and oral arms, by eliminating gastric contents followed by extensively washing the material with fresh water in order to maintain good quality and safety levels before processing to obtain a final product. These findings provide experimental data on the thousand-year old practice of JF pre-treatment in Asian Countries.

The use of optimized saline media is proposed here as an important emerging tool for going beyond the limits of conventional accredited methods for safety risk assessment of JF as human food. The present results, although preliminary, are in line with Commission Regulation (EC) N. 2073/2005 [21], where it is clearly expressed that the microbiological criteria already set are open to review and supplementing by considering “progress in science, technology and methodology, changes in prevalence and contamination levels, (…) as well as the possible outputs from risk assessments”.

## Figures and Tables

**Table 1:** Main safety and quality parameters identified for analysis of Jellyfish as a novel food.

Assays	Limits	Analytical Reference Method	Reference
Aerobic colony count	10^3.5^ < X < 10^5^ CFU/cm^2^ (for carcases cattle, sheep, goats, pigs and horses) 5 × 10^5^ < X < 5 × 10^6^ CFU/g (for minced and mechanically separated meat)	[22]	[14,34,35,36]
β-glucuronidase positive *Escherichia coli*	<10 CFU/g	[24]	[14,15]
Coliforms	<10 CFU/g ≤70 MPN/100 mL	[23]	[34,37]
Coagulase positive *Staphylococci*	10^2^ < X < 10^3^ CFU/g	[25]	[14,15]
*Salmonella* spp.	Absence/25 g	[28]	[14,15]
*Listeria monocytogenes*	Absence/25 g	[29]	[14,15]
Moulds and yeasts	<10^2^ CFU/g (Marinated octopus, seafood cocktail)	[26,27]	[35]
Histamine	100 < X < 200 mg/kg (fishery products) 200 < X < 400 mg/kg (fishery products treated in brine)	[31]	[14,15]
TVBN	<25–35 mg/100 g	[14]	[31,38]

**Table 2:** Safety and quality selected parameters applied to jellyfish (JF) and seawater samples following accredited conventional assays used for seafood and fish-derived products. Average of data obtained in four sampling periods (June, July, August and October 2018). Data were submitted to one-way analysis of variance. a,b—the different letters in line indicate significant differences among JF samples and seawater samples in the same assay (*p* < 0.05). ND—Not Detected.

Guideline	Media	Incubation Parameter	Jellyfish Samples			
			WJ	SJ	FWJ	C
			Microbiological Analyses			
			*CFU/g*			
Total bacteria count	PCA	30 °C for 72 h	5 × 10^1^–2.1 × 10^2^ (a)	2.7 × 10^1^–3.9 × 10^2^ (a)	1.7–8.5 × 10^2^ (a)	0–2.7 × 10^2^ (a)
Coliforms	ColiID Agar	37 °C for 24 h	<10 (a)	<10 (a)	0–7.7 × 10^2^ (a)	<10 (a)
*E. coli*	ColiID Agar	37 °C for 24 h	<10 (a)	<10 (a)	0–5.5 × 10^1^ (a)	<10 (a)
*Staphylococci*	Baird Parker Agar RPF	37 °C for 24 h	0–1.4 × 10^2^ (a)	0–3.7 × 10^2^ (a)	0–2.6 × 10^2^ (a)	0–1.5 × 10^2^ (a)
Yeast and Moulds	DRBC	30 °C for 72 h	<10 (a)	<10 (a)	<10 (a)	<10 (a)
			*CFU/25 g*			
*Salmonella* spp.	BPW	37 °C for 18 h	ND	ND	ND	ND
	RVS broth	41.5 °C for 24 h				
	MKTT broth	37 °C for 24 h				
*L. monocytogenes*	FBH	30 °C for 24 h	ND	ND	ND	ND
	ALOA Gelose Agar	37 °C for 48 h				
		*mg/kg*				
		Chemical analyses				
Histamine			<3	<3	<3	ND
			*mg/100 g*			
TVBN			0.22–1.39 (a)	0–1.39 (a)	3.51–5.56 (b)	ND

PCA: Plate Count Agar; RPF: Rabbit Plasma Fibrinogen; DRBC: Dichloran Rose–Bengal Chloramphenicol Agar; RVS: Rappaport Vassiliadis Salmonella; BPW: Buffered Peptone Water; MKTT: Mueller Kauffman Tetrathionate; FBH: Fraser Broth Half concentration.

**Table 3:** Bacteria, Yeast and Molds total counts obtained by JF and seawater samples using different saline media. Average of data obtained in four sampling periods (June, July, August and October 2018). a,b,c—the different letters in line indicate significant differences among JF samples and seawater samples in the same assay (*p* < 0.05).

Microorganisms	Media	Incubation Parameter	WJ	SJ	SFJ	C
			*CFU/g*			
Bacteria	sPCA Ny	30 °C for 72 h	2.5 × 10^2^–3.2 × 10^2^ (a)	1.1 × 10^2^–3 × 10^2^ (a,b)	2 × 10^1^–5 × 10^1^ (b)	9.5 × 10^2^–1 × 10^3^ (c)
	R2A Ny		2.2 × 10^3^–2.4 × 10^3^ (a)	1.5 × 10^3^–2 × 10^3^ (a,b)	2.5 × 10^2^–3.5 × 10^2^ (b)	1.3 × 10^3^–1 × 10^4^ (c)
	Marine Agar Ny		3 × 10^3^–4.5 × 10^3^ (a)	2.2 × 10^2^–1 × 10^3^ (b)	5 × 10^2^–6.2 × 10^2^ (b)	7.2 × 10^2^–9 × 10^2^ (b)
Yeast and Moulds	sCMA KA	30 °C for 72 h	9.8 × 10^1^–1.5 × 10^2^ (a)	3.5 × 10^1^–6.0 × 10^1^ (b)	0–2 (b)	2–5 (b)
	sSDA KA		1 × 10^3^–1.5 × 10^3^ (a)	4 × 10^1^–2.2 × 10^1^ (b)	1.6 × 10^1^–2 × 10^1^ (b)	1.2 × 10^1^–2 × 10^1^ (b)
	R2A KA		2.5 × 10^2^–3.2 × 10^2^ (a)	2.5 × 10^1^–3 × 10^1^ (b)	1 × 10^1^–2 × 10^1^ (b)	1 × 10^1^ (b)
	Marine Agar KA		3.6 × 10^2^–4 × 10^2^ (a)	2.8 × 10^1^–5.5 × 10^1^ (b)	1.5 × 10^1^–2.5 × 10^1^ (b)	1 × 10^1^–1.3 × 10^1^ (b)

sPCA: saline Plate Count Agar; Ny: Nystatin; KA: Kanamycin and Ampicillin; sCMA: saline Corn Meal Agar; sSDA: saline Sabouraud Dextrose Agar.
